# Negamycin: Nature’s Forgotten Antibiotic

**DOI:** 10.1021/acsinfecdis.5c00843

**Published:** 2025-12-26

**Authors:** Grant A. Boyle, Gregory S. Basarab

**Affiliations:** Holistic Drug Discovery and Development (H3D) Centre, University of Cape Town, Cape Town 7701, South Africa

**Keywords:** negamycin, antibacterials, total synthesis, structure-activity relationship, protein biosynthesis inhibition, ribosome, pharmacokinetics

## Abstract

Negamycin is a natural product antibiotic discovered in 1970 and shown to have a Gram-negative spectrum of activity. It has served as the starting point in drug discovery efforts due in large part to its structural simplicity and novel mode of inhibition of the bacterial ribosome. It follows that negamycin does not show cross-resistance with other antibacterial agents that operate on the ribosome, whether this would be due to target modification, drug efflux, or drug metabolism. Because of the deficiencies of current drug regimens for the treatment of infections caused by Gram-negative pathogens, having a new agent brought to the infectious disease formulary represents a critical medical need, as has been promoted by the World Health Organization and other entities. Negamycin has been the subject of over 20 total syntheses, often highlighting stereoselective chemistry toward installing its two chiral centers on an acyclic chain. Novel synthetic methodologies thereby developed can stimulate the synthesis of novel analogs. With this, progress has been made in devising more potent analogs than negamycin. Structural work has determined that negamycin binds to the A-site of the 30S ribosome encounter complex with tRNA. Advancements have been made to understand the mechanism of transport of negamycin to the bacterial cytoplasm to enable engagement of the ribosome. This review surveys much of what has been published around negamycin and its analogs, including aspects of the biological spectrum of activity and mode of action as well as limitations that have held back clinical development.

## Graphical abstract

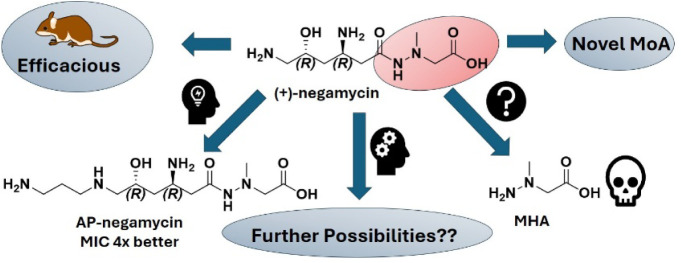

Natural products have important clinical utility in treating a variety of illnesses, the most prominent of which are those caused by bacterial infections.^1–5^ Though many natural products function without modification as therapeutic agents, they have also served as the starting point for new drug design initiatives.^6–8^ Nearly all current classes of antibiotics have offspring that have been derived by semisynthesis or by synthesis from non-natural precursors. This includes plazomicin among the aminoglycosides,^9^ cefiderocol among β-lactams,^10^ azithromycin among macrolides,^11^ rifampicin among ansamycins,^12^ tigecycline among tetracyclines^13^ and dalbavancin among glycopeptides.^14^ Key synthetic antibiotics without a natural product origin include sulfonamides,^15^ oxazolidinones^16^ and fluoroquinolones.^17^ Varying degrees of resistance have emerged from clinical use of all of these and other classes of antibacterials,^18–22^ leading to treatment failures and requiring diligent and difficult antibiotic stewardship to stave off further resistance development.^23^ Hence, it is worthwhile to reconsider natural product antibiotics that have not advanced clinically and for which there is no preexisting resistance. One such natural product is (+)-negamycin that exhibits a Gram-negative spectrum of antibacterial activity associated with life-threatening infections.^24–26^ Issues of resistance are particularly problematic in serious Gram-negative pathogens, where the search for new mode-of-action (MoA) agents is of paramount importance.^27^

In 1970, the structure of negamycin was elucidated after its isolation from a culture of *Streptomyces purpeofuscus* wherein higher antibacterial activity was disclosed against a variety of Gram-negative pathogens (including *Escherichia coli*, *Salmonella typhus*, *Klebsiella pneumoniae*, and *Pseudomonas aeruginosa*) and lower activity against Gram-positive pathogens.^28^ The higher Gram-negative activity perhaps gave rise to the compound name. Negamycin has a relatively low molecular weight (248.3) while being quite polar (logD is immeasurably low). It is not susceptible to bacterial efflux (at least for *E. coli* and *P. aeruginosa*)^29–31^ and shows a low frequency of resistance (FoR) for *E. coli* in M9 medium.^31,32^ Though population MICs across large collections of clinical isolates have not been disclosed, cross-resistance to established antibacterial agents has not been seen. Negamycin plasma protein binding is low (<16%) across species, and *in vivo* pharmacokinetics (PK) is favorable.^33^ Efficacy has been achieved in several animal models of infection.^28,32,34^ Over the decades, learnings around the negamycin MoA have revealed interactions with its target ribosome that lead to disruption of protein biosynthesis as have insights into how it is able to navigate through the bacterial envelope. The bacterial ribosome serves as the target of a large variety of clinically important antibiotics (tetracyclines, aminoglycosides, macrolides, oxazolidinones, and others) that have faced varying issues of resistance development that are not due, generally, to genetic alteration of the target code.^35,36^ Protein translation represents a complex process of initiation, elongation, termination, and recycling on a large (70S) ribosomal macrocomplex. Complex substrates (tRNAs, the growing peptide chain) are utilized, as are auxiliary proteins (Initiation factors: IF1, IF2, and IF3; elongation factors: ET-Tu, EF-G; termination factors: RF1 and RF2; release and recycling factors: RF3 and RRF).^37^ The net result is that there are multiple binding sites and multiple catalytic states that offer a plethora of mechanisms for the inhibition of protein synthesis. Notably, the mode of ribosome inhibition by negamycin differs from that of all other ribosome inhibitors, and there is no evidence that mechanisms resistant to established drugs (drug-modifying enzymes, post-transcriptional ribosome modification, ribosomal protection proteins, efflux) are applicable to negamycin.

Herein, we review much of what is known around negamycin, seeking to inspire further fundamental and applied research. We include current knowledge around the limitations of negamycin itself, among which are toxicological considerations that have perhaps precluded its development or the development of analogs toward a clinically useful drug. Toxicological concerns revolve around *in vivo* hydrolytic release of the right-hand side (RHS) methylhydrazine acetic acid (MHA) fragment, in line with pyridoxine deficiencies induced by hydrazines in general. There has been at least one reported analog with higher antibacterial activity than negamycin; further improvements in antibacterial activity would be in order to widen safety margins if indeed toxicity is associated with hydrolysis to MHA. Ultimately, there would be value in deriving a safe and effective negamycin-inspired antibacterial to treat antimicrobial resistance (AMR) due to the novel MoA relative to other antibacterials and the nonvulnerability to preexisting resistance.

## Total Synthesis

The total synthesis of negamycin has been an ongoing area of investigation for more than 50 years since its first discovery. The value in continuing to find new syntheses of negamycin includes the development and application of new methodology as well as the possibility of adapting the methodology to derive new analogs. In their very thorough digest paper,^38^ Zhu *et al.* describe several synthetic approaches to negamycin that had been published up to 2018.^39–57^ Herein, we focus on approaches that have been published since. Total syntheses of negamycin focus on the LHS setting up for late-stage amide formation with MHA followed by removal of protecting groups that might have been utilized. The chirality on the LHS has served as a focal point oftentimes for methodology development with either the chiral center at what becomes C5 directing chirality at C3^39, 43^ or vice versa.^40–42,47,49,51–53,55^ However, the most efficient syntheses (fewest steps, highest yields) have involved independent generation of each of the two chiral centers.^46,48,50,54,56,57^ Additionally, both chiral centers have been derived from natural product precursors^45^ and through chiral cycloadditions.^44^ As negamycin has a (+)-rotation, there has been interest in synthesizing its optical antipode as well as the other two possible diastereomers to investigate the structural requirements for activity and bacterial permeability (vide infra). To our knowledge, up to the review manuscript by Zhu *et al.*, the selective synthesis of (−)-negamycin with the opposite stereochemistry relative to the natural product had been reported in the literature only three times. The first was in 1972 starting from D-galacturonic acid^39^ and the second in 2014, wherein the known route established by Davies et al.^56^ for the synthesis of (+)-negamycin was adapted with reactants having the opposite optical antipode relative to (−)-negamycin.^32^ This route by Davies was also adapted to synthesize the 3*R*,5*S*- and 3*S*,5*R*-diastereomers (3-*epi*- and 5-*epi*-negamycin, respectively) of (+)-negamycin.^32^ Now, more recently, two novel approaches have been devised for the synthesis of (−)-negamycin, albeit not for the actual natural product with the (+)-rotation.

The first recent stereoselective synthesis of (−)-negamycin started from a key chiral epoxide **1** that was promoted by the authors for other purposes and that has five of the six carbons making up the main carbon backbone of negamycin (Scheme 1A).^58^ A diastereoselective iodocyclization set up the two chiral centers of **1** in line with C5 chirality directing C3 chirality. Copper-catalyzed ring opening of the epoxide with vinylmagnesium chloride followed by phosphorylation of the resultant alcohol with DPPA yielded diphenylphosphate ester **2** with concomitant desilylation of the terminal alcohol. The phosphonate and terminal alcohol were converted to azides by treatment with NaN_3_ and DPPA/DBU, respectively, to afford bis-azide **3**. Subsequent osmium tetroxide-catalyzed oxidative cleavage of the alkene yielded the corresponding acid **4** that was then coupled with benzyl (1-methylhydrazino)­acetate to obtain compound **5**. The final global deprotection step was carried out using high-pressure hydrogenolysis to yield the desired product, (−)-negamycin.

**Scheme 1:**
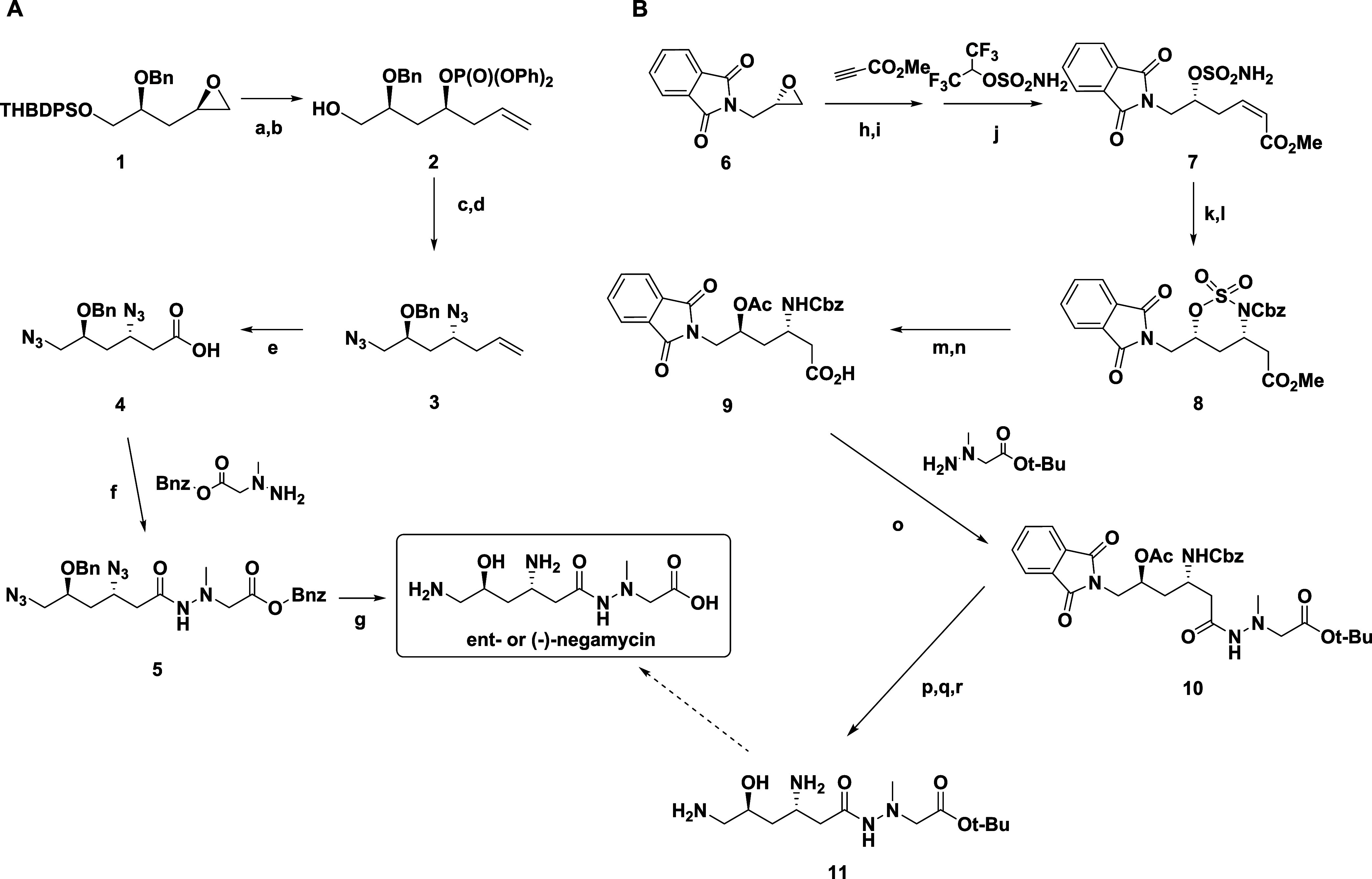
Recent Synthetic Approaches to (−)-negamycin^a^

a Reagents and conditions: (a) Vinylmagnesium chloride, CuI, 88%; (b) DPPA, DBU, 94%; (c) NaN_3_, DPPA, DBU, 95%; (d) TMSN_3_, DIAD, Ph_3_P, 73%; (e) OsO_4_, Oxone, 77%; (f) EDC, HOBt, Et_3_N, 68%; (g) H_2_, Pd­(OH)_2_/C, 93%; (h) n-BuLi, BF_3_.OEt_2_ (i) Lindlar’s catalyst, quinoline, H_2_, 93%; (j) Pyridine, 67%; (k) TBAF, 82%; (l) CbzCl, K_2_CO_3_, 79%; (m) KOAc, 79%; (n) Me_3_SnOH, 65%; (o) EDC, HOBt, Et_3_N, 76%; (p) K_2_CO_3_, 61%; (q) hydrazine hydrate, 64%; (r) H_2_, Pd/C, 62%.

In the second recent report, Joshi et al.^59^ applied a sulfamate-tethered diastereoselective *aza*-Michael cyclization methodology developed in their lab^60–62^ to the synthesis of the *tert*-butyl ester of (−)-negamycin (Scheme 1B). Again, C5 chirality directed the formation of C3 chirality. Starting with commercially available (*S*)-N-glycidylpthalimide **6**, regioselective epoxide opening with the anion of methylpropriolate was followed by Lindlar hydrogenation and Johnson-Magolan sulfamoylation^63^ to afford key intermediate **7**. This was cyclized to the sulfamate with TBAF in the *aza*-Michael reaction and acylated to the *Cbz* group to afford **8** with overall >20:1 diastereoselectivity. Ring-opening by heating **8** with KOAc and subsequent selective hydrolysis of the methyl ester via Nicolaou’s Me_3_SnOH methodology^64^ led to **9**. Amide formation with commercially available *tert*-butyl 2-(1-methylhydrazinyl)­acetate afforded **10**. The acetate group was removed using K_2_CO_3_, followed by cleavage of the phthalimide using hydrazine hydrate. Finally, hydrogenolysis of the *Cbz* group yielded the desired (−)-negamycin *tert*-butyl ester **11**. However, though seemingly simple to do, the conversion of the *tert*-butyl ester of **11** to the carboxylic acid was not reported in the manuscript, nor is it known in the open literature.

## SAR Discussion

In order to discuss where changes have been made to the molecule during SAR investigations, negamycin was broken down into the LHS and RHS parts of the molecule (hexanoate and MHA, Figure 1). SAR investigations have been carried out by several different laboratories with antibacterial activity being determined in various ways (zones of inhibition vs MIC determinations) and in various media that lead to quantitatively different values. For example, Table 1 shows MICs generated for negamycin across a variety of *E. coli* strains and medium conditions. Though it is sometimes easier to describe the activity of analogs relative to that seen for negamycin, herein determined MIC values along with the medium used for the determinations will be generally presented. In addition to antibacterial activity, several labs reported activity against an *E. coli* cell lysate transcription-translation (TT) assay measuring disruption of protein biosynthesis. TT IC_50_’s will be included when disclosed in the literature.

**Figure 1:**
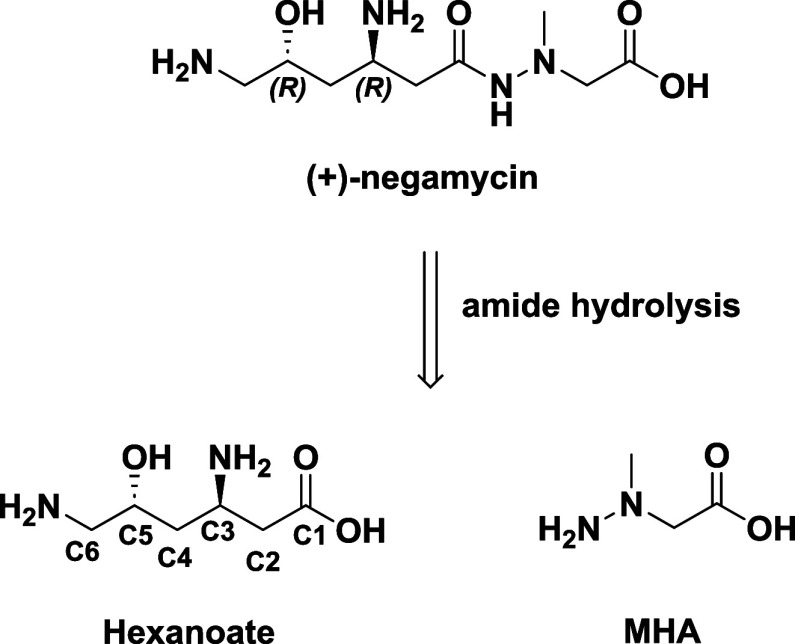
Structure of negamycin and amide hydrolysis subunits, the negamycin hexanoate and methylhydrazine acetic acid (MHA).

**Table 1:** MICs (μg/mL) for Ngamycin against Various *E. coli* Strains in a Variety of Media^a^

	**MHB** ^32^	**MHB** ^31^	**MHB +50% Hu serum** ^67^	**MHB2** ^30^	**M9** ^31^	**MHA** ^68^	**M9 + thia** ^31^	**M9 + glu** ^32^	**LB** ^31^	**PP water** ^31^	**PP water +10–40% serum** ^28^	**PP Ag.** ^28,39,70^	**Nu Ag.** ^66^
**ATCC25922**	16	>64	4	32	—		2	4	>64	8			
**BW25113**	64	—	—	—	4				64	16			
**K-12**	—	—	—	—	—						1.56–3.12	1.56–3.12	3.12
**K-12 ML1629**	—	—	—	—	—							1.56	6.25
**NIHJ**	—	—	—	—	—							3.12	12.5
**MG1655**	—	—	—	16	—			2					
**BEM11**	—	—	—	—	—	2							

a MHB = Mueller–Hinton Broth; MHB2 = cation-adjusted MHB; M9 = minimal medium; MHA = Mueller–Hinton Agar; M9 + glu = M9 + glucose; LB = lysogeny broth; M9 + thia = M9 + thiamine; PP = 0.5% peptone; PP ag = PP in agar; Nu. Ag = nutrient agar.

Over the years, there have been nearly 20 published total syntheses of (+)-negamycin and about six reports directed at analog SAR investigations. All four diastereomers of negamycin have been individually synthesized and isolated toward evaluating the stereochemical influence on activity.^32^ The natural negamycin (*R*,*R*)-stereochemistry affords the highest activity both for inhibition of the ribosome measured in the *E. coli* TT assay, and for antibacterial activity using fully rich Mueller-Hinton Broth medium, which might be expected if natural design selects for activity. The opposite antipode of negamycin (*S*,*S*) showed nearly 5-fold lower activity in the *E. coli* TT assay and 16-fold or higher MICs against the pathogen. The other two diastereomers showed even lower target and antibacterial activities. However, in minimal M9 media supplemented with glucose, the 3*S*,5*S* opposite enantiomer and 3*S*,5*R* epimer of (+)-negamycin were nearly equipotent to (+)-negamycin against *E. coli*. This has been ascribed to greater recognition for active transport by the inner membrane transporter dipeptide-binding protein A (dppA) for the 3*S-*configured negamycin diastereomers versus compounds with the 3*R* configuration.^32^ As with any antibacterial agent, activity is a function of target potency and bacterial cell envelope permeability, which will be discussed in more detail later.

## RHS Hydrazide Modifications

As diagrammed in Figure 1, the RHS hydrazide could be envisioned to undergo *in vivo* hydrolysis releasing (*3R*,*5R*)-3,6-diamino-5-hydroxyhexanoic acid (hexanoate) and MHA, the latter bringing a concern of potential toxicity (see below).^65^ In early studies, Umezawa *et al.* found that the negamycin terminal carboxylate was important for activity against *E. coli* with the corresponding carboxamide **12a** (Figure 2) being 4- to 7-fold less active.^66^ This result was later corroborated when **12a** was resynthesized and shown to have greatly diminished activity in the *E. coli* TT assay in addition to the lower activity against the pathogen itself.^30^ Similarly, carboxylic acid isosteres such as tetrazole **12b**, phosphonic acid **12c,** and phosphinic acid **12d** were not tolerated.^67^ Replacing the carbon for the hydrazide nitrogen *α*-to the carboxylate (**12e**) led to 16-fold lower activity than negamycin against *S. aureus* with no data provided against Gram-negative pathogens.^41^ Replacing the same nitrogen with oxygen (hydroxamate ester **12f**) also led to significantly lower activity.^30,67^ Removal of the hydrazide methyl group (**12g**) or replacing it with ethyl (**12h**) significantly diminished activity.^30^ Placing a methyl group on the carbon *α*- to the acid (**12i**) led to 2- to 8-fold lower antibacterial activity relative to negamycin depending on the pathogen.^30^ It is possible that one of the diastereomers would contain the activity and therefore afford a 1- to 4-fold reduction in activity. However, an ethyl group on the *α-*carbon (**12j**), again as a diastereomer mixture failed to show antimicrobial activity under the test conditions.^30^ Addition of an extra carbon into the backbone to form **12k** was not tolerated.^66^ Neither was *N*-methylation of the carboxamide nitrogen (**12l**).^30^ An attempt to tie the hydrazide portion of negamycin into a pyrrolidine ring (**12m** and **12n**) also eliminated activity.^67^ The net conclusion that can be made is that the carboxylate and hydrazide functionalities are required for activity, as is the NH of the hydrazide. The carboxylate has been modeled to interact with Mg^2+^ in several *E. coli* ribosome-negamycin complexes (see below), while the hydrazide carbonyl, perhaps in the imidyl tautomer due to the higher acidity of the NH, binds to a backbone phosphate, consistent with the essentiality of the pharmacophores.^57^ As the changes to the RHS hydrazide portion of the molecule have not been well-tolerated, the aforementioned concerns of potential toxicity due to negamycin hydrolysis releasing MHA have not yet been alleviated.

**Figure 2:**
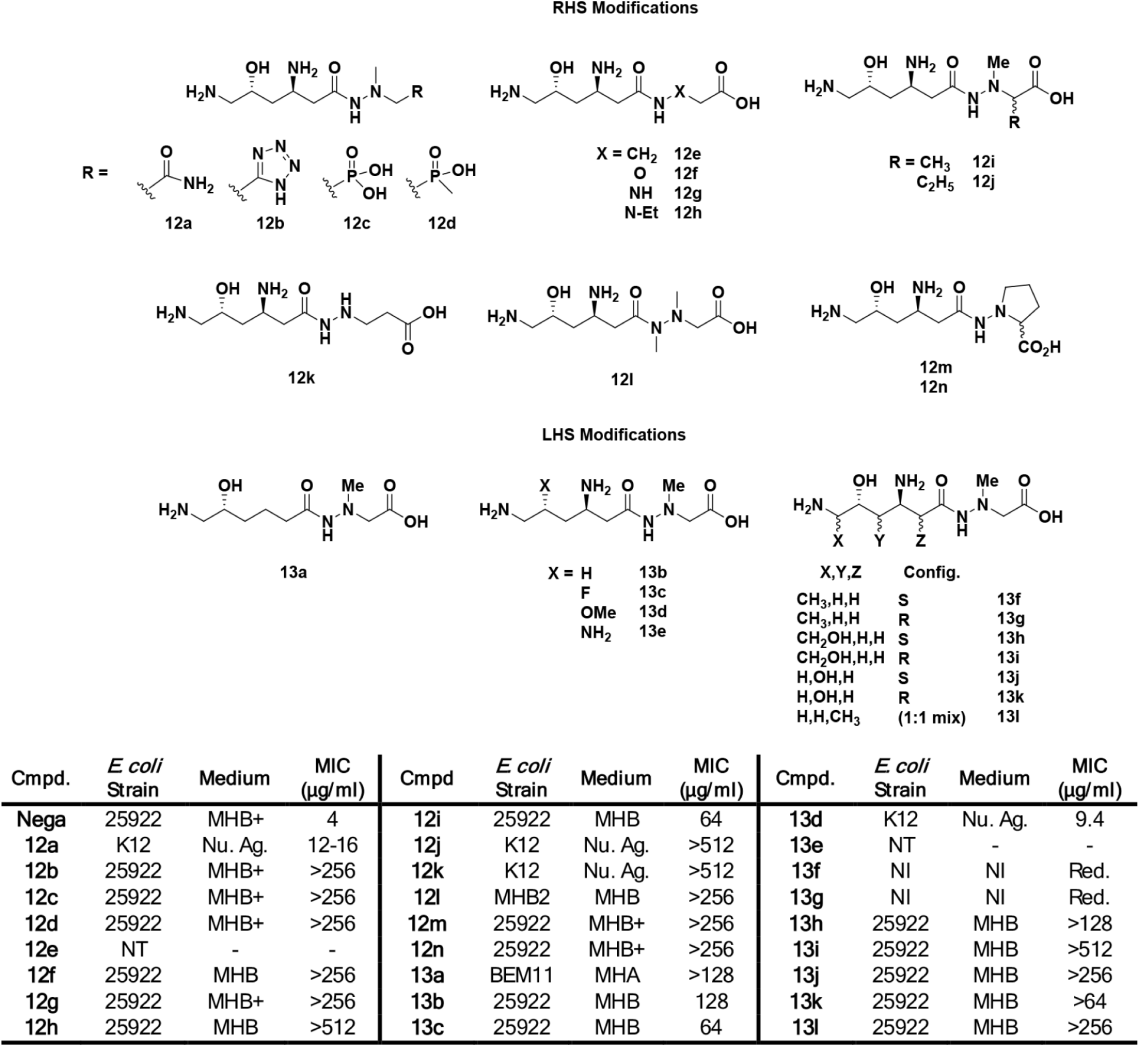
MICs resulting from RHS and LHS modifications of negamycin (Nega) in various media: Nu. Ag. = nutrient agar; MHB = Mueller–Hinton Broth; MHB+ = MHB plus 50% human serum; MHA = Mueller–Hinton Agar; NT = not tested; NI = not indicated; Red. = reduced activity relative to negamycin.

## C3 Amino Group Modifications

The only negamycin analog wherein the C3 amino group was modified has been its replacement with hydrogen (**13a**, Figure 2), which led to no antibacterial activity at the highest concentrated tested.^68^ This amino group was modeled to make a key salt bridge to a backbone phosphate as delineated by X-ray crystallography studies,^57,69^ accounting for its importance to activity.

## C5 Hydroxy Group Modifications

Derivatives at the negamycin C5 position have been prepared, wherein the hydroxyl was replaced with H, F, NH_2_, and OCH_3_ (Figure 2). Replacing the hydroxyl with a hydrogen (**13b**) atom lowered activity 2- to 4-fold in the *E. coli* TT assay and against Gram-negative pathogens.^30,34,70–72^ Replacing the hydroxyl group with a fluorine atom (**13c**, maintaining the *R*-stereochemistry) lowered activity in the *E. coli* TT assay by only 1.7-fold and lowered *E. coli* antibacterial activity 4-fold.^30^ This perhaps demonstrates a negative influence on bacterial permeability for the fluorine atom due to the lowering basicity of the two amines. Having a fluorine atom in the *S*-configuration lowered activities 10-fold or greater relative to the *R*-configuration.^30^ Capping the hydroxyl with a methyl group (OCH_3_ derivative **13d**) lowered antibacterial activity 3-fold.^66^ Replacing the hydroxyl with NH_2_ (**13e**) led to no activity at the highest concentrations tested.^41^ The results overall showed that the hydroxyl group at C5 can be altered, but the alterations have decreased activity.

## Substitutions on C2, C4, C5 of LHS Hexanamide Chain

There have been several analogs made with substitutions along the LHS carboxamide chain of negamycin (Compounds **13f**-**13l**, Figure 2) with the resultant compounds generally showing significantly lower activity than negamycin.^30^ In one exception, **13k** with the 4-position hydroxyl in the *R*-configuration displayed only a 2-fold lower potency in the *E. coli* TT assay relative to negamycin. However, it showed an MIC against *E. coli* over 8-fold higher in line with lower basicity for the C3 amine compromising bacterial permeability.

## Modifications of the Negamycin 6-Amino Substituent

Soon after the report of negamycin’s isolation in 1970, Kondo *et al.* reported the isolation of leucyl-negamycin (**14c**) wherein the C6 amino group of negamycin was acylated with leucine.^73^ As a parenthetic note, it was suggested (without supporting data) that leucyl-negamycin is the biosynthetic precursor to negamycin. The data indicated a measure of operability for derivatization of the amino group and led to a further exploration of substituents on the C6-amine of negamycin. In 2004, leucyl-negamycin was synthesized, and antibacterial activity was shown to be diminished 2- to 16-fold relative to negamycin.^67^ Hence, a collection of negamycin analogs derivatized on the C6-amine were made and evaluated (Table 2).^67^ Notably, two compounds (**14d** and **14e**) displayed higher inhibitory potencies than negamycin in the *E. coli* lysate TT assay yet showed lower antibacterial activity, presumably due to less optimal bacterial permeability. The medium for the antibacterial assays included 50% human serum, which apparently leads to lower MICs, at least for *E. coli*, relative to no added serum. In another observation, relative antibacterial activity generally tracked across pathogens in that as activity diminished against, for example, *E. coli*, it also diminished against the other pathogens, both Gram-negative and Gram-positive (data for the latter not shown).

**Table 2:**
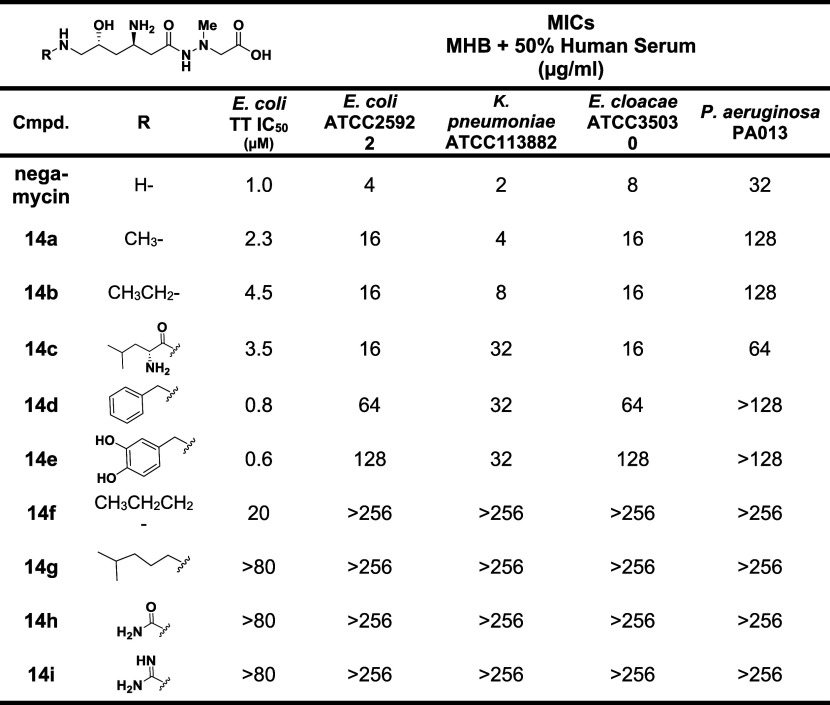
Activity of C6-Amine Derivatives of Negamycin—1st Report^67^

In a second report delineating further elaboration of C6 amine substituents, higher antibacterial activity than negamycin was achieved for the first time (Table 3).^30^ Notably, **15a** with an aminopropyl substituent on negamycin showed a 5-fold lower IC_50_ in the *E. coli* TT assay relative to negamycin as well as 2- to 8-fold lower MICs across the Gram-negative bacterial pathogens. The branched N-aminobutyl derivative **15b** (mixture of diastereomers) showed equipotent activity to **15a** in the biochemical and antibacterial assays, if it is assumed that activity predominantly resides in one of the diastereomers. The alternate branched-chain aminobutyl derivative **15c** also showed about equal activity to negamycin, again if the activity predominates in one of the resultant diastereomers. Several other C6 amine substituents showed higher or comparable activity in the TT assay to negamycin; however, diminished antibacterial activity was observed. Among these are derivatives with N-aminobutyl (**15d**), *N*-hydroxyethyl (**15i**), N-guanidinylpropyl (**15o**) and 3-amino-2-hydroxypropyl (*R*-isomer,**15g**). Again, as previously indicated, subtle changes in compound charge and lipophilicity speak to bacterial permeability deviations that can influence the expression of MIC values. Retrospective analysis of negamycin-ribosome crystal structures points to an expanded binding region around the terminal C6 negamycin amine that explains the operability of the substituents.^57,69^ A cryo-EM structure of the *E. coli* ribosome in complex with the aminopropyl derivative of negamycin (**15a**) afforded a binding model wherein the terminal amine forms a salt bridge with a backbone phosphate, thereby accounting for its improved activity over negamycin.^74^

**Table 3:**
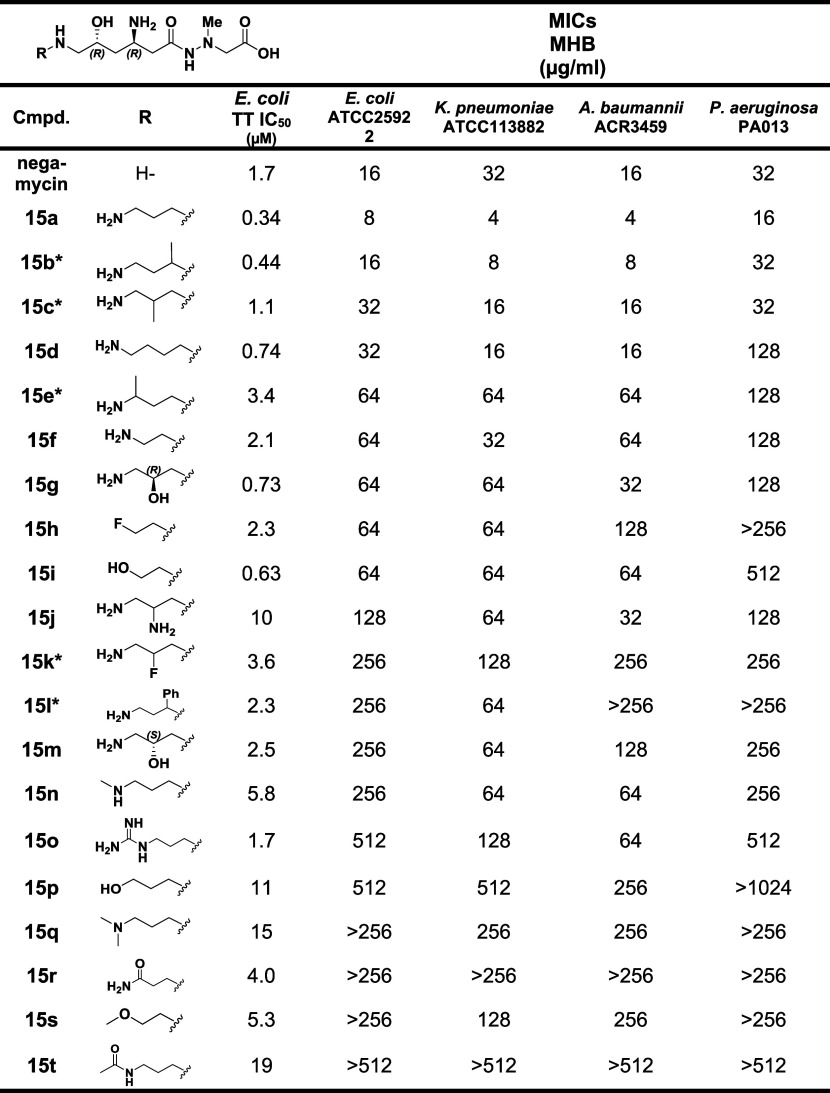
Activity of C6-Amine Derivatives of Negamycin—2nd Report^30^

Several alternatives to the N-propyl amines were made (Figure 3), in part, to rigidify the flexible N-terminal chain. Compounds **16a**–**c** were about 2- to 6-fold less potent than negamycin in the *E. coli* TT assay and at least 16-fold less active in the *E. coli* MIC assay.^30^ Compounds **16d**–**g** were described as having a drastic loss in antibacterial activity, indicating a need to maintain the C6 substituent as either a secondary or primary amine.^67^

**Figure 3:**
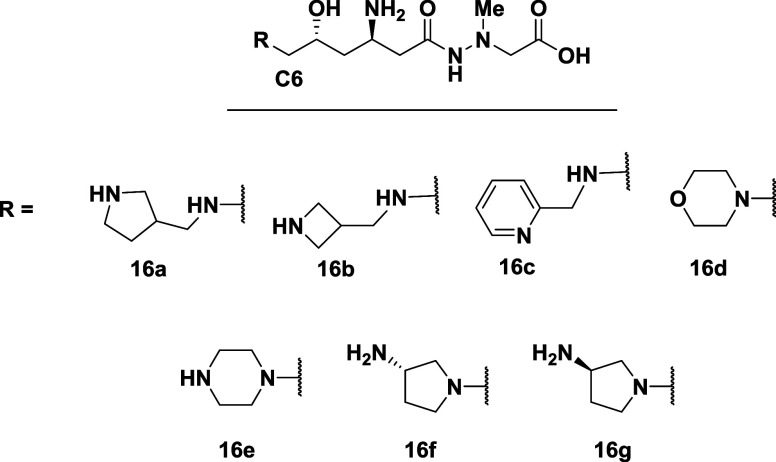
Alternative C6-amine substituents: MICs ≥ 256 μg/mL against *E. coli*.

## Conformationally Restricted Modifications of Negamycin

Raju *et al.* reported the synthesis and SAR evaluation of a series of conformationally constrained deoxynegamycin derivatives, focusing exclusively on the C4 to N-terminal portion of the negamycin backbone.^34^ The structures of the key compounds are listed in Figure 4. Most of the compounds were significantly (>10-fold) less active than negamycin in both the *E. coli* TT assay and in *E. coli* MIC determinations. One of the diastereomers (undetermined) of compound **17g** showed equipotent activity to negamycin in the TT assay; however, it failed to register an MIC against *E. coli* at the highest concentration tested (thereby showing >32-fold lower antibacterial activity). Again, the results reinforce the need to optimize bacterial permeability as well as target potency.

**Figure 4:**
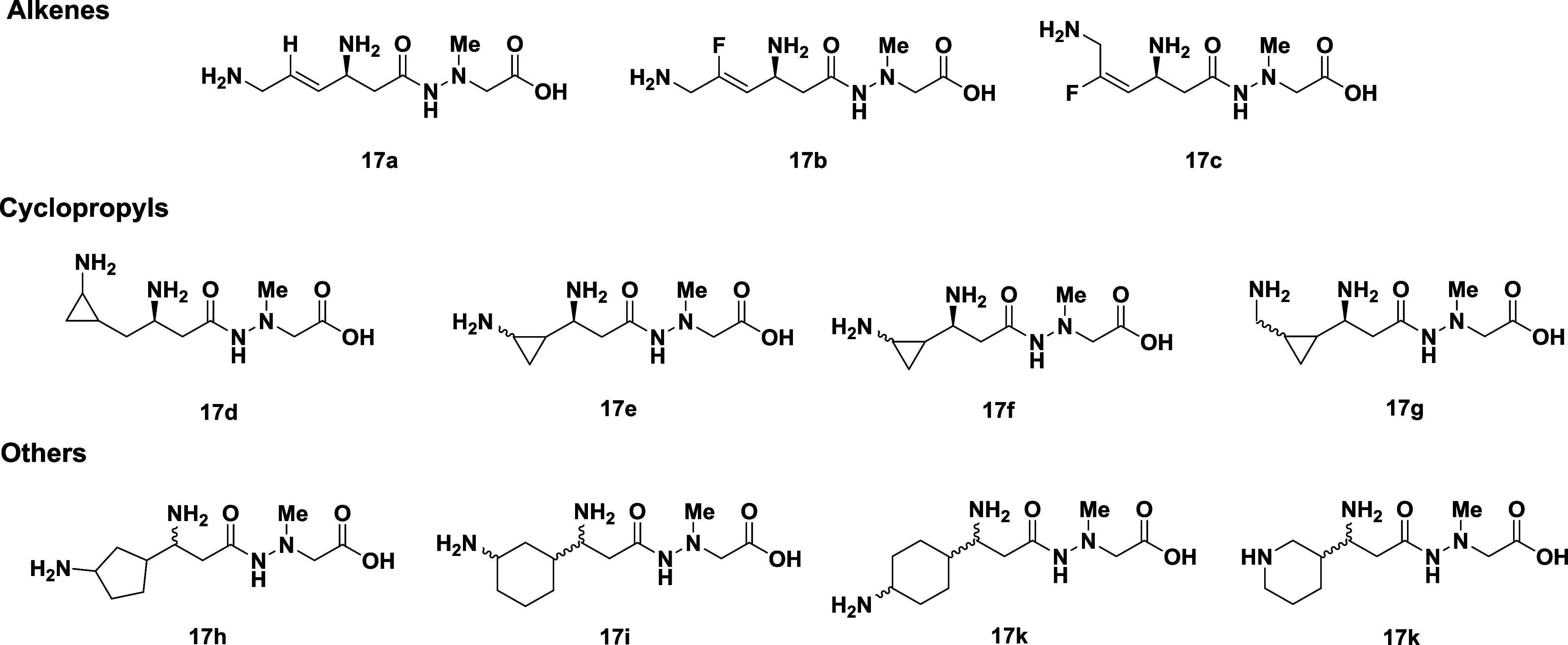
Conformationally restricted negamycin analogues: MICs ≥ 256 μg/mL against *E. coli*.

## Readthrough Activity

While not the focus of this review, it would be remiss to neglect investigations into negamycin analogs as eukaryotic readthrough agents for the potential treatment of human genetic diseases, of which Duchenne Muscular Dystrophy (DMD) has been primarily investigated. This disease is caused by mutations in the DMD gene, including a subset of nonsense mutations leading to premature termination codons (PTC’s) during protein synthesis. The DMD gene encodes dystrophin, an essential protein for the formation of the muscle cell skeleton. A chemotherapeutic approach for the treatment of this disease would involve readthrough agents inducing the ribosomal machinery to skip PTC’s during translation, thereby delivering full-length functional proteins. Hayashi and co-workers synthesized and evaluated various negamycin analogs with activity for DMD readthrough. Minimal antibacterial activity (when delineated) was observed for the analogs, demonstrating a divergence from readthrough SAR.^68,75–78^ Compounds **18a**–**d** (Figure 5) have been highlighted for DMD readthrough; compound **18d** (TCP-306) showed readthrough activity nearly 15-fold higher than negamycin and over 2-fold better than the best aminoglycoside (G418, a relative of gentamicin), a class also demonstrating eukaryotic readthrough activity.

**Figure 5:**
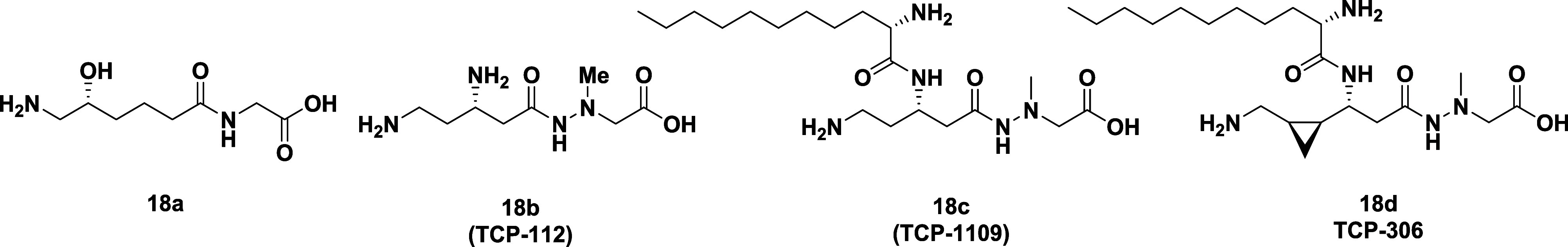
Readthrough agents.

## Negamycin Mode-of-Action

Soon after its isolation was described, two reports from the Japanese National Institute of Health reported that negamycin operates by disrupting protein synthesis in *E. coli*. More specifically, two mechanisms were invoked: the inhibition of protein synthesis initiation^79^ and the promotion of mRNA misreading, leading to aberrant protein synthesis,^29^ the latter being a characteristic also seen in aminoglycoside antibiotics. Inhibition of protein synthesis was demonstrated by negamycin blockage of ^14^C-amino acid incorporation in *E. coli* culture during exponential growth. Using *E. coli* cell lysate, it was also shown that negamycin blocked the incorporation of ^14^C-amino acids into protein from an exogenous mRNA template. More recently, also using *E. coli* cell lysate, an IC_50_ = 1.8 μM was determined for negamycin in the TT assay, which compared favorably with the tetracycline IC_50_ = 1.5 μM. Notably, there is considerable overlap of the tetracycline binding site with that of negamycin based on structural work (see below).^57^ The binding of ^14^C-labeled formylated methionyl-tRNA (met-tRNA_F_) to an mRNA-ribosome complex was impeded by negamycin, implicating disruption of the A-site of the 30S ribosome subunit where protein synthesis is initiated (see Figure 6).^79^ Incorporation of other macromolecular ^14^C-labeled precursors (glucosamine, adenine) was much delayed, discounting peptidoglycan and DNA biosynthesis as MoA’s. Incorporation of ^3^H-CMP by DNA-dependent RNA polymerase was not impeded by negamycin, negating inhibition of RNA synthesis.

**Figure 6:**
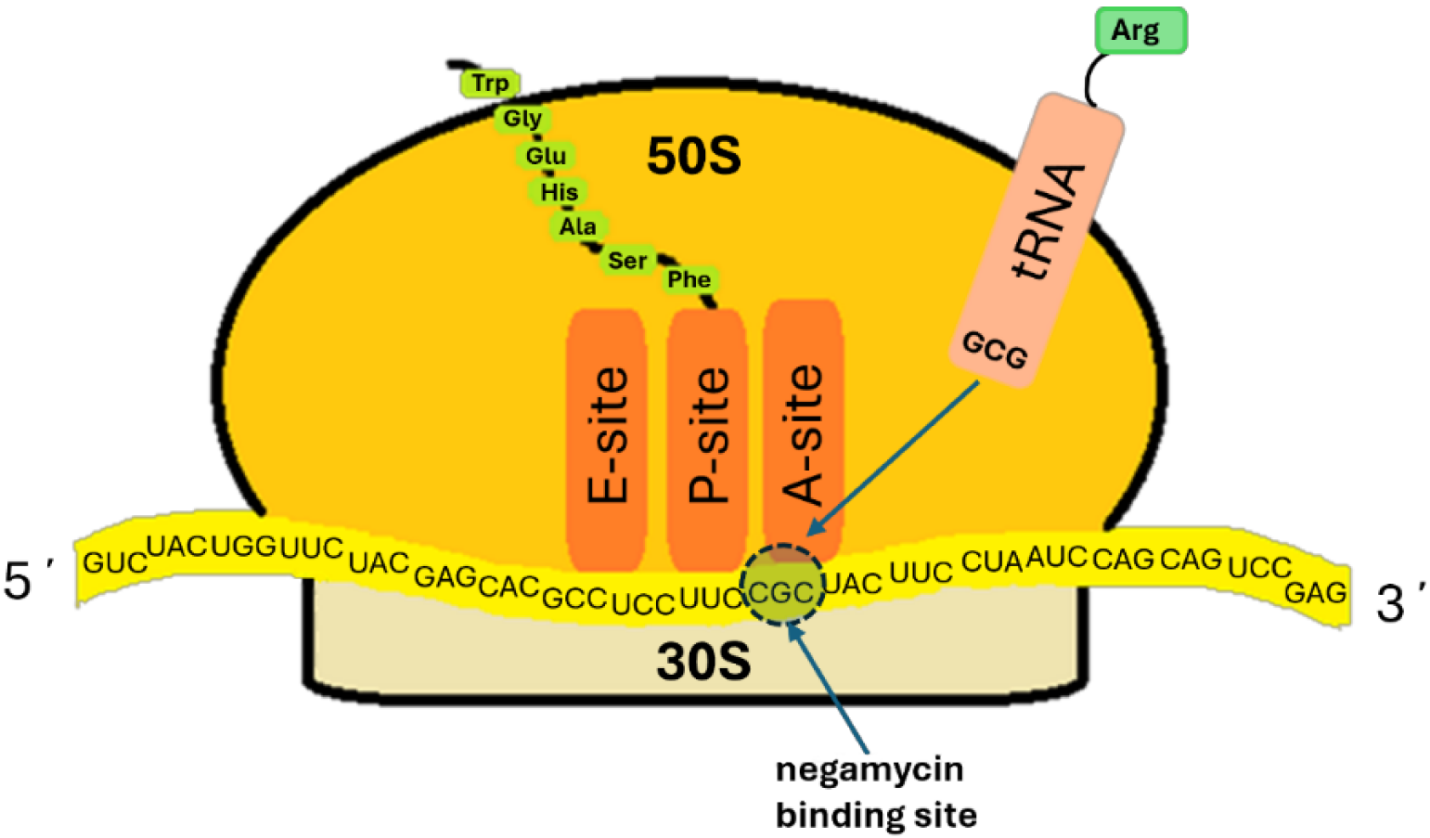
Diagram of the ribosome delineating the negamycin binding site on the A-site of the 30S subunit where tRNA binds.

Misreading, the secondary negamycin MoA, was demonstrated by negamycin-induced incorporation of leucine (most notably, but also isoleucine and serine) from an artificial poly-uracyl mRNA template during the production of polyphenylalanine in *E. coli* lysate. Using artificial polyadenine and polycytosine mRNA templates, measures of glutamate and leucine incorporation, respectively, were also observed in polylysine and polyproline production, respectively. However, the nature of this misreading was shown to differ from that seen for aminoglycoside-resistant ribosomes that were shown to be equally sensitive to negamycin, and the respective binding sites were hypothesized to differ at the time,^29^ which has since been confirmed. Misreading activity was also demonstrated using a luciferase gene construct harboring a stop codon wherein negamycin (and streptomycin but not tetracycline) induced a measure of readthrough.^31^ More recent work using single-molecule Förster resonance energy transfer (smFRET) methods showed that negamycin, even at high concentrations (>100-fold over the IC_50_), did not affect the selection of cognate fluorescence-labeled Phe-tRNA^Phe^.^57^ This stands in direct contrast to tetracycline, which blocks binding of the cognate aa-tRNA from the recognition site. Using a near-cognate mRNA template in the assay did not preclude binding of the labeled Phe-tRNA^Phe^ in the presence of negamycin as opposed to doing so in its absence. It follows that the binding of near-cognate aa-tRNA could progress to aberrant protein biosynthesis.^57^

Additionally, negamycin has been shown to impede the release of ribosome-bound peptides, which has also been observed for aminoglycoside antibiotics.^80,81^ RNA-directed protein synthesis experiments monitoring the incorporation of ^14^C-labeled amino acids demonstrated a higher accumulation of ribosome-bound peptides in the presence of negamycin. Overall, the characterization of the negamycin MoA becomes quite complicated due to multiple correlated processes being interrupted.

Mapping of ribosome inhibition has been investigated by resistance generation studies coupled with genetic sequencing to determine if disruptions with the ribosome A-site correlate with negamycin activity. Work from AstraZeneca, Weill Cornell Medical College, and UC Berkeley documented the selection of negamycin-resistant mutants against *E. coli* SQ110,^57^ a strain engineered with only one genomic copy of the gene (*rrn)* encoding the ribosome rather than the natural complement of seven.^82^ Whole-genome sequencing of resistant clones identified a unique mutation (U1052G) located on the 16S rRNA adjacent to Helix 34 (h34), encompassing the aa-tRNA binding site and the negamycin binding site, as later delineated by structural work (see below). The mutants had MIC values 8-fold higher relative to those against the parent SQ110 strain, which correlated well with negamycin showing an IC_50_ 4.5-fold higher for the U1052G ribosome construct than that of wildtype in TT assays using purified recombinant components.^83^ Furthermore, negamycin’s capability to promote near-cognate recognition and, therefore, misreading was significantly lowered with the U1052G construct, as seen in smFRET experiments.^57^ Notably, the U1052G mutant strain was 4-fold hypersensitive to tetracycline, in line with overlapping binding with negamycin. That the tetracycline MIC shift was target-based was supported by the tetracycline IC_50_ being 5.5-fold lower against the U1052 mutant than that seen against the wildtype ribosome in TT assays.^83^ Mutant selection against negamycin was also carried out against the genomic *rnn*-null *E. coli* SQ171 transformed with a plasmid-encoded *rrn*C operon leading to 4-fold lower sensitivity and to the identification of U1060A, a nucleotide also near the negamycin binding site.^57^ Overlap of the binding site between tetracycline and negamycin was supported by displacement of ^3^H-labeled tetracycline from the *E. coli* 70S ribosome on titration with negamycin and by crystallography work (see below).^57^ Since tetracycline resistance in the clinic is not associated with ribosome mutations due to the higher copy of the *rrn* gene encoding the ribosome, target-based cross-resistance between negamycin and tetracycline would not be expected and is not seen.^57^ Independently (and published 2 days later), research work from Yale University and the University of Illinois detailed the selection of resistant mutants against a hyperpermeable (Δ*tolC*) *E. coli* SQ110 strain.^69^ Resistant clones were found only on the 16S rRNA gene: U1052G, U1060A, and A1197U. A1197 is also associated with the 30S ribosomal decoding center base-pairing with U1060. Plasmids with the U1060A and A1197U mutations were introduced into a hyperpermeable Δ*tolC rnn*-null *E. coli* SQ171 strain, leading to 4-fold and 16-fold reductions in negamycin sensitivity relative to the introduction of the wild-type plasmid. Hence, the relevancy of the negamycin binding site to activity was established by these two complementary studies.

## Structural Biology

The negamycin binding site on the bacterial ribosome and insights into the mode of inhibition have been delineated in several X-ray crystallography and cryo-EM investigations. As would be expected due to the dynamic nature of ribosome function, no one snapshot of negamycin bound to various ribosome constructs will capture the entire process of inhibition and misreading. In 2007, researchers at Yale University reported the crystal structure of the *Haloarcula marismotui* 50S ribosome (PDB 2QEX) wherein negamycin in complex with a metal ion was modeled to be bound to the peptide exit tunnel.^84^ This may be an artifact of the crystallography work relative to the primary negamycin mode of inhibition as delineated by the aforementioned studies implicating the 30S ribosome A-site, wherein the met-tRNA_F_ approach was blocked and spontaneously generated RNA mutations were observed. Subsequent X-ray crystallography work using the entire 70S ribosome from *Thermus thermophilus* and *E. coli* has placed negamycin at the A-site alongside h34 interfacing otherwise with the tRNA substrate (see Figure 6). Neither the *T. thermophilus* nor the *E. coli* crystal structures showed electron density that corresponded to that seen within the peptide exit tunnel in the *H. marismotui* 50S ribosome structure.

The *T. thermophilus* ribosome complex with negamycin included mRNA and deacylated tRNA, wherein three molecules of the latter were seen, one each in the A, P, and E sites (Figure 6).^69^ As mentioned, whereas negamycin interfaces with tRNA, tetracycline blocks its approach to the A-site. Due to a large excess of negamycin being used, putative electron density was observed at nine different sites with only one located at a ribosomal functional center, namely, that of A-site. The other eight sites are thought to involve electrostatic attractions that might be expected between dibasic negamycin and polyanionic ribosomal RNA. In the A-site, the negamycin carboxylate ligates an Mg^2+^ directly and the hydrate of a second Mg^2+^ (Figure 7A). The carbonyl is positioned to interact with a bridging phosphate oxygen of G1197, and the terminal amine bridges phosphate oxygens of G_966_ and A_965_. Two nucleotides of the tRNA (U33 and G34) are associated with the complex via interactions with the phosphate hydrate.

**Figure 7:**
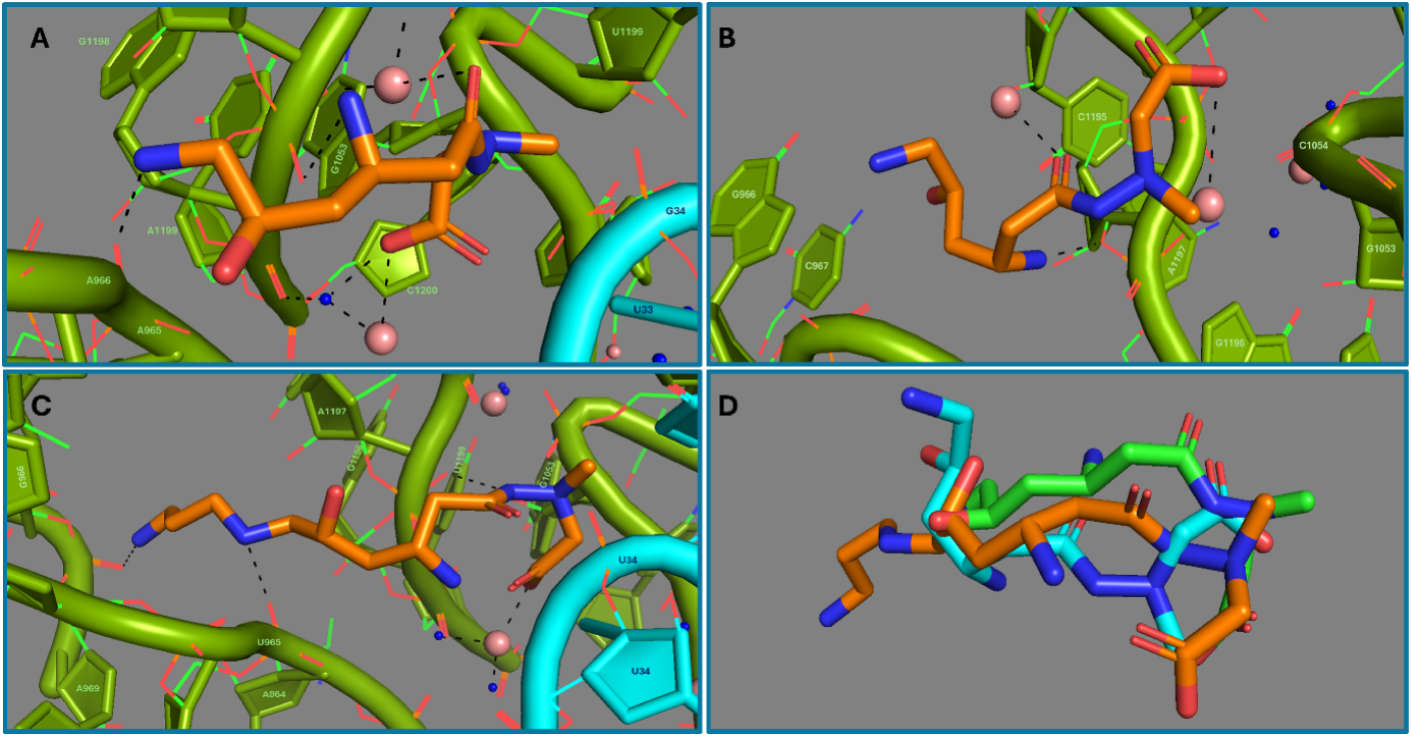
Negamycin structural work. 16s rRNA is colored green; Mg^2+^ ions are shown as rose spheres; waters are shown as blue dots. **A.** (PDB 4W2I) X-ray crystallographic model of negamycin (orange carbons) at the interface of the *T. thermophilus* ribosome A-site and deacylated tRNA (cyan). The negamycin carboxylate and carbonyl groups are proximal to Mg^2+^ ions. **B.** (PDB 4WF1) X-ray crystallographic model of negamycin (orange carbons) at the *E. coli* ribosome A-site. The negamycin carboxylate and carbonyl groups are proximal to Mg^2+^ ions. **C.** (PDB 7B5K) Cryo-EM model of compound **15a** (orange carbons) at interface of the *E. coli* ribosome A-site and suppressor tRNA (t1A3T3, cyan). The terminal amine of compound **15a** interacts with the bridging phosphate of A969. **D.** Overlay of negamycin from the *T. thermophilus* and *E. coli* ribosome X-ray crystal structures (green and cyan carbon atoms, respectively) and **15a** (orange carbons) from the *E. coli* cryo-EM structure derived by alignment of key 16S rRNA nucleotides of the 30S ribosome.

The X-ray crystal structure of negamycin bound to *E. coli* 70S ribosome showed the inhibitor interfacing with the phosphate backbone of C1054, A1197, and G1198, and, as indicated earlier, nearby the resistance determinants , U1052 and U1060 (Figure 7B).^57^ The negamycin binding site delineated by the *T. thermophilus* and *E. coli* structures overlaps that previously elucidated for tetracycline antibiotics. In the A-site, a Mg^2+^ hydrate bridges nonbridging phosphate oxygens of the 16s rRNA U1054 and A_1197_ to negamycin. The A1197 nonbridging phosphate oxygen also can accommodate a hydrogen bond with the negamycin carboxylate. The 3-amino group of negamycin can form H-bonds and/or salt bridges with nonbridging phosphate oxygens of G1198 and U965 as well as a contact with a bridging phosphate oxygen of A1197. The terminal amine of negamycin is situated close to the two nonbridging phosphate oxygens of G966.

In 2021, researchers from the University of Hamburg and Uppsala University solved via cryo-EM an *E. coli* 70S ribosome structure in complex with an ErmCL (erythromycin-dependent leader peptide) UGA stop codon construct, a suppressor tRNA, and **15a**, the aminopropyl derivative of negamycin.^74^ The ErmCL construct, in the presence of erythromycin, induces ribosome stalling and positions a UGA stop codon for tRNA recognition. The suppressor tRNA (designated t1A3T3) was specifically designed and optimized to decode the UGA stop codon. Compound **15a** was included to block the stop codon interaction with the tRNA. In the cryo-EM structure (PDB entry 7B5K), **15a** binds to both the 16S rRNA of the A-site and the anticodon step loop of the tRNA (Figure 7C). The 3-position amine and the carboxylate of **15a** approach one another to form an intramolecular H-bond/salt bridge. The 3-position amine and the carboxamide carbonyl of **15a** engage nonbridging phosphate oxygens of U34 and U35, respectively, of the tRNA. Compound **15a** is positioned to interact with the nonbridging phosphate oxygens of G1198 of 16S rRNA via the hydrazide nitrogen atoms, while the carboxylate of **15a** ligates a Mg^2+^ ion that reaches over to nonbridging phosphate oxygens of A964 and U1199 via water hydrates. The 6-position amine of **15a** forms a salt bridge with nonbridging phosphate oxygens of U965 and G966. Importantly and accounting for the higher activity of **15a**, the terminal amino group is positioned to form a salt bridge to nonbridging phosphate oxygens of A969 and an H-bond to the ribose hydroxyl of A968. The structure accounts well for the recognition of tRNA constructs at the ribosome A-site by negamycin.

The X-ray crystallography and cryo-EM-derived structures account for the suggested multiple modes of disruption of protein synthesis in that negamycin stalls initiation of protein synthesis and enables the recognition of noncognate tRNA to induce misreading. The structures may represent different states of the ribosome relative to catalysis and substrate (e.g., tRNA) binding. An overlay of the three ribosome structures via alignment of surrounding nucleotides from 16S rRNA shows close correlation of the binding site. Negamycin and **15a** assume different conformational states and interactions with the surrounding residues (Figure 7D). This, of course, discounts the possibility that the fit to the electron density has a measure of uncertainty. However, it emphasizes that the disruption of ribosomal function is a dynamic phenomenon that adds considerable complexity to detailed inhibitory mechanisms and ribosome interactions.

## Microbiological Properties

The spectrum of *in vitro* antimicrobial activity of negamycin comprises serious Gram-negative pathogens including Enterobacteriaceae (*E. coli*, *Salmonella* spp., *K. pneumoniae*, *Shigella* spp., *Proteus vulgaris*, *Providencia rettgeri*), and nonfermenters (*Acinetobacter baumannii*, *P. aeruginosa*).^28,30^ Negamycin MICs are unchanged against isogenic *E. coli* strains wherein tolC, acrA, acrB, or acrAB has been knocked out supporting that it is not a substrate for efflux pumps.^30,31^ As well, MICs were maintained against an isogenic efflux-debilitated *P. aeruginosa* strain (*mexABCDXY* ^*‑*^) relative to the wild-type strain.^30^ MICs against Gram-positive pathogens (*Staphylococcus aureus*, *Streptococcus pneumoniae* and *Bacillus subtilis*) range from 4- to 16-fold higher than those for Gram-negatives.^28,39,67^ As mentioned, MICs against *E. coli* vary depending on medium conditions (see Table 1). The addition of serum to cultures may lower MICs (compare values against *E. coli* ATCC299 in MHB with and without serum), in line with the proposal that serum bicarbonate lowers MICs of polybasic compounds. This has been ascribed to the disruption of the bacterial cytoplasmic membrane and the proton motive force^85^ and might, therefore, be applicable to negamycin. The caveat is that the experiments with and without serum were not carried out side-by-side in a single laboratory. Additionally, it is unclear which medium would best correlate to bacterial growth *in vivo* and be most relevant to PK/PD analyses.^86^ *In vitro*, negamycin was shown to have a bactericidal, irreversible killing action.^80^ Negamycin and its analogs diverge from trends to identify precision antibacterials with perhaps a single pathogen spectrum of activity;^87–89^ negamycin analogs would also fall short as pure empiric therapies that seek coverage for both Gram-positive and Gram-negative pathogens. Nonetheless, they could find utility for early empiric administration to treat severe infections when Gram-negative infections are suspected or diagnosed.^90,91^

Overall, *in vitro* *E. coli* data support a low propensity for spontaneous resistance development on exposure to negamycin. In M9 at 4-fold over the MIC against *E. coli*, negamycin demonstrated a FoR <7 × 10^–9^, in line with the pathogen’s high copy number (7) for the ribosome gene if target-based resistance were anticipated.^31^ At 1- and 2-fold the MIC, the FoR climbed to about 10^–7^ with mutations seen in inner membrane transport machinery; notably, the fitness of the mutants was compromised suggesting that they would not be manifested in the clinic.^31,32^ In fully rich MHBII using the *E. coli* SQ110 strain to select for target-based mutations as mentioned above, a low FoR = 10^–9^ was seen at 4-fold over the MIC, with mutants reflecting alterations of h34 of the 16s rRNA.^57^ High FoR’s ranging from 10^–6^ to 10^–7^ were seen at 1- and 2-fold over the MIC;^57^ however, all recovered mutants were compromised in fitness (see below).

A complicating factor for quantitating negamycin antibacterial activity is the variability reported in the literature arising from different assay and medium conditions. Indeed, this variability was specifically addressed across a series of media showing negamycin *E. coli* MICs ranging from 2 μg/mL in M9 to 8 μg/mL in PP (0.5% polypeptone) to >64 μg/mL in MHB (Mueller–Hinton broth) and LB (lysogeny broth).^31^ Previously, against the identical *E. coli* strain (ATCC25922) showed MICs = 32 and 4 in MHBII and M9/Glu media, respectively.^30^ At the very least, this variation is in line with the sensitivity of negamycin to the nutrient peptide content in the media (see below). Shifts in MICs with media variations were also seen for other bacterial pathogens. Additionally, it was shown that MICs vary with the added cations and cation concentrations. For example, the negamycin MICs against *E. coli* in PP shifted from 8 μg/mL with no added cation to 2 μg/mL with 2.5 mM CaCl_2_ to 64 μg/mL with 64 mM NH_4_Cl. That Ca^2+^ might affect negamycin MICs correlated with measurable (albeit weak) cocomplexation as determined by isothermal calorimetry. That the shift in MICs due to Ca^2+^ is not related to target affinity was demonstrated by its lack of effect on the negamycin IC_50_ in the TT assay.^31^ The pH of the medium can also influence MICs as demonstrated by negamycin having an MIC = 8 in unbuffered PP (pH = 7), an MIC = 64 at pH = 5, and an MIC = 2–4 at pH = 8.5. That this would be the case may relate to the electrical potential across the inner membrane, where the pH is higher in the cytoplasm, as has been seen for aminoglycosides that are similarly net positively charged. Higher uptake at more basic pH values was supported by accumulation studies with tritiated negamycin, wherein uptake increased 53% at pH = 8.5 relative to pH = 7.^31^

## Bacterial Permeability

Negamycin is unusual relative to most antibacterials in that it has a small molecular weight (248 g/mol), very low lipophilicity (logD < −1) and high solubility (essentially miscible with water). Its high activity against Gram-negative pathogens begs the question of how it traverses the bacterial cell envelope to reach the cytoplasm and target the ribosome. Indeed, though there are a plethora of viable bacterial targets for drug intervention,^92,93^ compound capability to enter the cell is oftentimes limited by the contradictory requirements for traversing the outer and inner membranes.^95^ To understand permeability, resistant mutants of an *E. coli* strain (W3110) were raised to negamycin in minimal (M9) media, in which it showed an MIC = 4 μg/mL. Following 1- and 2-fold concentrations of negamycin relative to its MIC in M9, whole genome sequencing of resistant colonies showed mutations in the *dpp* operon including several in the *dppA* gene and a 100 kb deletion of the entire *dppABCDF* operon.^32^ Similar studies to generate resistant mutants to negamycin in another *E. coli* strain (BW25113) yielded 12 colonies all of which contained aberrations of the *dpp* operon, the most predominant of which involved the operon being entirely deleted.^31^ The dppA protein (and others in the operon) is an ATP-dependent inner membrane transporter thought to enhance the delivery of short (2–8 residue) nutrient peptides to the cytoplasm. One of the *dppA* mutants (with an I409 insertion) led to an 8-fold increase in the negamycin MIC to 32 μg/mL in M9/Glu medium. The interpretation is that dppA recognizes negamycin and aids in its transport to the cytoplasm. A higher negamycin MIC (32 μg/mL) was seen using fully rich cation-adjusted Mueller–Hinton broth (MHBII) medium and likely reflects competition by dipeptides and other nutrients for dppA thereby blocking negamycin transport. This was supported by titration with l-Ala-l-Ala (up to 100 μM) to wild-type *E. coli* in M9 increasing the negamycin MIC to 32 μg/mL. No change in MIC was seen by titration of the Δ*dppA* mutant.^31^ To correlate the MIC shifts to bacterial permeability, it was shown that tritiated negamycin uptake in Δ*dppA* *E. coli* in M9 (in these experiments with added thiamine) was lowered by 20% after 60 min relative to the isogenic wild-type strain.^31^ Perhaps surprisingly, the Δ*dppA* *E. coli* mutant with the I409 insertion was shown to colonize mice on intramuscular inoculum injection, almost equal to that seen for wild-type *E. coli*.^32^ Hence, the mutation did not greatly prevent proliferation both *in vitro* and *in vivo*. Whether the mutation affects the broader array of nutritional peptide uptake was undetermined. A much broader range of oligopeptide transporter deletions in isogenic *E. coli* strains were assessed to identify several other transporters (dppB, dppC, dppD, and dPPF) with identical MIC shifts in M9 to that seen for dppA.^31^ It follows that negamycin is recognized by multiple oligopeptide transporters. In contrast, *E. coli* mutants with deletions in amino acid transporters (lysP, hisP, cadB, hisQ, hisJ, argT, and hisM) did not affect negamycin MICs and do not likely contribute to its transport.^31^

Though peptide transporters were thus implicated in shepherding negamycin into the cytoplasm, other permeability mechanisms must also operate to account for MICs seen in fully rich media. In MHBII using *E. coli* SQ110, resistant colonies harboring mutations in *cpxA*, *hem* genes, and *ubi* genes were identified by culturing negamycin at concentrations of 1- and 2-fold relative to MIC values followed by whole genome sequencing.^32^ All of the genetic mutations are known to maintain a proper membrane potential in the bacterium and have been linked to aminoglycoside resistance generated in the lab. As would be expected, such mutations compromise bacterial viability and have not been seen in the clinic as a cause of aminoglycoside resistance. A set of respiratory chain mutations (Δ*ndh*, Δ*sdhA*, Δ*ubiG*, Δ*ubiI*, Δ*ubiX*) responsible for maintaining the inner membrane potential from the *E. coli* Keio collection of single-gene knockouts^94^ showed 2- to 4-fold increases in MICs over wildtype in MHBII but little shift in M9.^31^ Furthermore, the negamycin MIC increased 4- to 8-fold under anaerobic conditions, where the membrane potential is also altered, an effect also seen for aminoglycosides.^31^ The data support a hypothesis that the membrane potential is largely responsible for negamycin transport when oligopeptide transporters are non-operable.

The permeability work for negamycin points to the Gram-negative inner membrane limiting permeation to the cytoplasm. By extension, it can be inferred that negamycin readily and passively traverses the bacterial outer membrane with its lipopolysaccharide component and the periplasmic peptidoglycan layer, although there are no studies supporting this. One might speculate that the peptide-like structural motif of negamycin enables entry through the outer membrane via porins that enable permeation of nutrient oligopeptides.

## ADME and PK

The initial manuscript on negamycin isolation reported (without experimental detail) that intramuscular injection of 50 mg/kg of the compound to a rabbit gave a serum concentration of about 100 μg/mL after 1 h with 80% excreted in the urine at 24 h. A high concentration (4480 μg/mL 1–2 h post-administration) of negamycin in urine was also observed.^28^

More recent characterization of cross-species ADME and PK showed negamycin to have quite favorable attributes as a potential IV agent.^33^ Considerable development work was needed to detect negamycin from a biological matrix to support such studies given its high hydrophilicity and dibasic/monoacid functionality. Ultimately, a novel nonafluoropentanoic acid carrier increased the negamycin retention time for reverse-phase HPLC analysis to enable quantitation. Plasma protein binding (PPB) ≤16% was measured for mouse, rat, dog, and human across negamycin concentrations spanning two orders of magnitude. Hepatocyte clearances were also low (≤1 mL/min/kg in rodents and 2.0 and 2.3 mL/min/kg in dog and human, respectively). On IV administration, steady-state volumes of distribution (Vd_ss_) were low (0.5–0.6 L/kg) as were plasma clearances (Cl_p_ =11.5, 8.7, and 3.4 mL/min/kg for mouse, rat, and dog, respectively). Negamycin *T*_1/2_ values were 2.1, 2.6, and 7.9 h, respectively. With the low negamycin PPB, unbound *in vivo* clearances (Cl_u_) are therefore also quite low. The main route of clearance *in vivo* proved to be renal, with unchanged negamycin (90–100%) found in the urine. Allometric scaling, PBPK modeling, and single-species (dog) scaling led to quite favorable predicted human Cl_p_ ranging from 2.5–2.8 mL/min/kg (average Cl_u_ = 3.0 mL/min/kg) and a Vd_ss_ ranging from 0.5–0.6 L/kg. The predicted negamycin half-life in human was 9.5–11.6 h, which compares favorably with values seen for broad-spectrum Gram-negative IV antibacterials such as ciprofloxacin, meropenem, and gentamicin (∼4, 1, and 2.5 h, respectively).

As hydrolysis of negamycin *in vivo* could release MHA as a metabolite, methods to analyze the latter were developed.^33^ No MHA was detected in plasma across mouse, rat, and dog PK experiments; 0.05% and 0.25% MHA were detected in the urine of rat and dog, respectively. Though the amount of MHA that could be recovered was very low, there is nonetheless concern that it could be responsible for the toxicity (see below).

The PK data overall correlate with the highly hydrophilic nature of negamycin, wherein it is minimally susceptible to metabolism and cleared renally. The predicted human clearance for negamycin is quite low,^33^ and its potential to treat urinary tract infections might be supported.

## Efficacy in Animal Models of Infection

The initial manuscript describing the isolation and characterization of negamycin also described efficacy studies wherein negamycin was administered IP in mouse models of *P. aeruginosa*, *K. pneumoniae*, *Salmonella* *typhosa* and *Staphylococcus aureus* infections, wherein lethality CD_50_’s of 4.4, 5.0, 2.4, and 12.5 mg/kg, respectively, were observed.^28^ Details were not included around experimental procedures, *in vivo* drug concentrations achieved, or MIC values against the particular bacterial strains used in the experiments. Hence, it is difficult to correlate the pharmacodynamics results with PK. More recently, an *E. coli* thigh infection model in the mouse showed a favorable dose response on q4 administration of 100–600 mg/kg (total doses). The 600 mg total dose (100 mg/kg every 4 h) showed a 6.5-log reduction in CFU counts relative to the untreated control and a 2.5-log reduction relative to stasis.^32^ Hence, negamycin was shown to be a cidal agent *in vivo*.

## Toxicology

There is relatively little published information on negamycin (and analog) toxicological effects. From an *in vitro* perspective, the high solubility of negamycin and **15a** enabled their evaluations at quite high (1 mM) concentrations against secondary pharmacology targets to survey potential toxicological issues. Neither showed appreciable inhibition against 58 secondary pharmacology targets encompassing common enzymes, ion channels, and GPCRs.^30^ This includes an IC_50_ > 1 mM against the hERG ion channel, which is the bane of many drugs and drug candidates. Additionally, neither showed activity in several cell-based assays, including cytotoxicity (THLE cells) or drug-induced phospholipidosis. Negamycin did register an IC_50_ = 555 μM monitoring contractions in iPSC cardiomyocyte cells. Overall, the off-target profile is favorable.

IV administration of negamycin was reported to show LD_50_ = 400–500 mg/kg in mice. IP administration of 200 mg/kg for 10 days showed no observed toxicity.^28^ Daily administration of negamycin to dogs caused reversible hepatic coma that halted clinical progression according to a report.^65^ No experimental details for the dog study were revealed including data around the dose, dosing regimen (including days of dosing), drug exposure, and time to reversibility, as well as the nature of the hepatic encephalopathy. It is unknown what the therapeutic window might be and whether the toxicity was associated with hydrolysis of the negamycin carboxamide to release MHA, as was suggested. MHA, having been detected in the urine of rats and dogs in trace amounts, was mentioned as being an inhibitor of glutamate pyruvate transaminase, a pyridoxal phosphate-utilizing enzyme, which would be in line with hydrazone formation with the cofactor aldehyde, the substrate pyruvate, or the product α-ketoglutarate. Increased levels of the transaminase and other pyridoxal phosphate enzymes in response to certain drugs have been correlated to liver tissue damage and hepatotoxicity,^96^ though it is unclear whether inhibition would also lead to liver damage.

As mentioned, negamycin induces a measure of misreading as part of its MoA in prokaryotic cells, but it also does so in eukaryotic cells, which might bring the concern of mammalian toxicity. It would therefore be prudent to understand the SAR associated with the readthrough activity of the eukaryotic ribosome to mitigate potential toxicological liabilities in the design of negamycin-based antibacterial agents.

## Summary

Antibacterial development has been decidedly weak without a truly novel MoA action drug targeting Gram-negative pathogens entering the marketplace since the 1960s.^97^ Much of this is due to the daunting hurdles around bacterial permeability and the difficulty of translating target-based discovery work into viable drugs. As well, bleak market prospects have discouraged the pharmaceutical industry from making the necessary commitment to discover and develop new antibacterials.^97,98^ This is despite a predicted medical crisis that will intensify if the effectiveness of current antibacterial continues to wane, suggesting that, to society, the value of novel antibiotics should be inordinately high. Within this context, negamycin, with its novel mode of inhibition of the bacterial ribosome, is differentiated from all other antibacterial agents, including other ribosome inhibitors. The negamycin binding to the ribosome A-site in association with aa-tRNA is distinguishing from tetracyclines that also bind to the A-site but block the approach of aa-tRNA. This ternary complex between negamycin, aa-tRNA, and the ribosome can lead to mistranslation of the mRNA template, producing aberrant proteins, which also contribute to the observed antibacterial activity. Resistance to tetracyclines arises from mechanisms other than mutations in the target site; however, negamycin is not cross-resistant to such mechanisms, nor is it cross-resistant to other ribosome-inhibiting antibacterials, much less other antibacterial agents. This includes ribosomal protection protein-based resistance (tetM) to tetracyclines and tetracycline metabolic resistance (TetX).^57^ Hence, identification of a negamycin analog with favorable PK and toxicology properties, in addition to favorable antibacterial activity would be a welcome addition to the formulary. Through the years, only a few analogs of negamycin, namely, those with an aminopropyl substituent on the terminal nitrogen, have shown higher antibacterial activity. The aminopropyl substituent (of **15a**) is thought to extend to a distal phosphate backbone and ribose hydroxyl to account for its higher target potency; importantly, the substituent does not seem to affect bacterial permeability relative to negamycin. Data have supported that bacterial permeability, as for any antibiotic, contributes to the expression of MICs and needs to be optimized in conjunction with target potency. Investigations into understanding bacterial permeability have indicated that the inner lipid membrane serves as the primary barrier to negamycin reaching the cytoplasm and interacting with the ribosome. The membrane potential largely enables negamycin to pass through, much as it does for aminoglycosides, which is important given that the polar, charged nature of the compound does not lend itself to passive diffusion through lipid bilayers. In addition, it has been shown that dipeptide transporters, such as dppA can ferry negamycin across the inner membrane under appropriate culture conditions. The spectrum of antibacterial activity for negamycin centers around serious Gram-negative pathogens for which there is a clear and present clinical need. The PK properties of negamycin are favorable in terms of plasma and urine concentrations. Negamycin has been validated by mouse models of infection, including thigh infections with *E. coli*.

More work is needed to identify negamycin analogs with improved activity against targeted pathogens, perhaps showing MICs ≤ 1 μg/mL in a culture medium indicative of *in vivo* efficacy. Extensive profiling of analogs should be carried out against large panels of drug-resistant clinical strains to demonstrate limited preexisting reduced susceptibility. Of course, the favorable PK profile of negamycin would need to be maintained in a potential clinical candidate toward achieving a reasonable human dose (perhaps <1 g per day). However, a potential liability exists for the negamycin hydrazide functionality that has thus far been essential for antibacterial activity. The key concern is that *in vivo* hydrolysis of the hydrazide would lead to MHA, a potentially toxic (although not yet demonstrated) metabolite. It is the aspiration of this review that it will inspire continued work around negamycin to attain improved compounds that would have favorable clinical utility in treating infections caused by serious Gram-negative pathogens.

## Abbreviations:

aa-tRNA: (aminoacyl-tRNA)
ADME: (absorption, distribution, metabolism, and excretion)
AMR: (antimicrobial resistance)
CbzCl: (benzyl chloroformate)
Cl_p_: (plasma clearance)
cryo-EM: (cryogenic electron microscopy)
CLSI: (Clinical and Laboratory Standards Institute)
DBU: (diazabicycloundecene)
DIAD: (diisopropyl azodicarboxylate)
DMD: (Duchenne Muscular Dystrophy)
DPPA: (diphenyl phosphoryl azide)
dppA: (dipeptide-binding protein A)
EDC: (3-(ethyliminomethyleneamino)-N,N-dimethylpropan-1-amine)
ErmCL: (erythromycin-dependent leader peptide)
FoR: (frequency of resistance)
h34: (Helix 34 of 16S rRNA)
HOBt: (hydroxybenzotriazole)
IC_50_: (half-maximal inhibitory concentration)
IP: (intraperitoneal)
LD_50_: (median lethal dose)
LHS: (left-hand side)
M9: (minimal medium)
met-tRNA_F_: (formylated methionyl-tRNA)
MHA: (methylhydrazine acetic acid
MHB: (Mueller-Hinton Broth)
MIC: (minimum inhibitory concentration)
MoA: (mode-of-action)
mRNA: (messenger RNA)
*n*-BuLI: (*n*-butyllithium)
PK: (pharmacokinetics)
PPB: (plasma protein binding)
PTC: (premature termination codon)
PP: (polypeptone)
RHS: (right-hand side)
SAR: (structure-activity relationship)
RND: (resistance-nodulation-division)
smFRET: (small molecule Förster resonance energy transfer)
TBAF: (tetrabutylammonium fluoride)
TMSN_3_: (trimethylsilyl azide)
tRNA: (transfer RNA)
TT: (transcription-translation)
V_ss_: (volume of distribution at steady state
